# Renal Cell Carcinoma with Clear Cell Papillary Features: Perspectives of a Differential Diagnosis

**DOI:** 10.1007/s12253-019-00757-3

**Published:** 2019-10-26

**Authors:** Áron Somorácz, Levente Kuthi, Tamás Micsik, Alex Jenei, Adrienn Hajdu, Brigitta Vrabély, Erzsébet Rásó, Zoltán Sápi, Zoltán Bajory, Janina Kulka, Béla Iványi

**Affiliations:** 1 2nd Department of Pathology, Semmelweis University, Üllői út 93, Budapest, H-1091 Hungary; 2 Department of Pathology, University of Szeged, Szeged, Hungary; 3 1st Department of Pathology and Experimental Cancer Research, Semmelweis University, Budapest, Hungary; 4 Department of Urology, University of Szeged, Szeged, Hungary

**Keywords:** Clear cell carcinoma, Clear cell papillary carcinoma, Cytokeratin 7-positivity, Differential diagnosis, *VHL* gene

## Abstract

Thirty-one cases of low-grade renal cell carcinoma (RCC) with clear cells and tubulopapillary/papillary architecture were analyzed retrospectively with immunohistochemical and genetic markers to gain more experience with the differential diagnosis of such cases. All samples coexpressed CK7 and CA9; the TFE3 or TFEB reactions were negative; the CD10 and the AMACR stainings were negative in 27 cases and 30 cases, respectively. The FISH assays for papillary RCC, available in 27 cases, and deletion of chromosome 3p, available in 29 cases, gave negative results. The results for 3p deletion, *VHL* gene mutation or *VHL* gene promoter region hypermethylation testing, along with the diffuse CD10-positivity in 2 cases confirmed 21 cases as clear cell papillary RCC (CCPRCC; CK7+, CA9+; no 3p loss, no *VHL* abnormality) and 10 cases as clear cell RCC (CCRCC; CK7+, CA9+; no 3p loss, *VHL* abnormality mutation/hypermethylation present). In CCPRCCs, the representative growth pattern was branching tubulo-acinar, commonly accompanied by cyst formation. The linear nuclear arrangement or cup-shaped staining of CA9 did not necessarily indicate CCPRCC, and the absence of these did not exclude the diagnosis of CCPPRC. One tumor infiltrated the renal sinus; the others exhibited pT1 stage; and metastatic outcome was not recorded. The CCRCC cases were in pT1 stage; 6 exhibited cup-shaped staining of CA9, and 1 displayed lymph node metastasis at the time of surgery. Distant metastatic disease was not observed. In summary, the *VHL* abnormalities distinguished the subset of CCRCC with diffuse CK7-positivity and no 3p loss from cases of CCPRCC.

**Electronic supplementary material:**

The online version of this article (10.1007/s12253-019-00757-3) contains supplementary material, which is available to authorized users.

## Introduction

Clear cell papillary renal cell carcinoma (CCPRCC) is an infrequent subset of RCC [1, 2]. Although CCPRCC shares histopathogical features with clear cell RCC (CCRCC), papillary RCC and Xp11.2 translocation RCC, its immunohistochemical coexpression of cytokeratin 7 (CK7) and carbonic anhydrase 9 (CA9), and negativity for CD10, alpha-methyl-CoA racemase (AMACR), and TFE3 usually clarifies the diagnosis [3–7]. The renal angiomyoadenomatous tumor (RAT) is now regarded as being in the spectrum of CCPRCC [8–10]. Genetically, CCPRCCs lack chromosome 3p deletion or *VHL* gene mutation or *VHL* promoter hypermethylation, the hallmarks of CCRCC, and have no loss of chromosome Y or gain of chromosome 7 and 17, the hallmarks of papillary RCC [2–4, 11–13].

In surgical pathology practice, the separation of CCPRCCs from CCRCCs can pose certain difficulties. The distinction is crucial, because CCPRCCs have a very limited potential for metastasis (fatal outcome has been reported only in two patients out of 400 [14]), whereas in low-grade CCRCCs distant metastases can occur several years after nephrectomy. To learn more about the differential diagnosis of low-grade RCCs with CCPRCC features, a series of such tumors were subjected to a retrospective immunohistochemical analysis, applying CK7, CA9, CD10, AMACR, TFEB and TFE3 immunostainings, and the immunophenotypes were correlated with the results of genetic markers for CCRCC or papillary RCC.

## Materials and Methods

### Case Selection and Review Process

This study was conducted with the permission of the Medical Research Council (17489-4/2017/EKU). The hematoxylin and eosin-stained slides of 2326 consecutive RCC samples were reexamined for clear cell papillary RCC-like tumors, including low-grade nuclei, the presence of any degree of tubulopapillary growth pattern of tumor cells with clear cytoplasm, linear arrangement of nuclei away from the basal membrane, along with the presence of a leiomyomatous stroma. Demographical and clinical data were collected from the database management systems of Semmelweis University and University of Szeged.

### Tissue Microarray and Immunohistochemical Reactions

Tissue microarray blocks were prepared for immunohistochemistry with TMA Master (3DHISTECH) applying a 2 mm core diameter. One to four representative cores were then punched out from the donor blocks. Immunohistochemical staining for CA9, CK7, CD10, AMACR, TFEB and TFE3 were performed (see the dilutions and sources in Supplementary Table 1). The epitope retrieval was performed for each antibody according to the manufacturer’s recommendations. The reactions were conducted using Autostainer (Dako). Afterwards, slides were evaluated microscopically by estimating the proportion (%) of immunopositive cells. Staining in over 50% of the tumor cells, in 10 to 50% of tumor cells, or in less than 10% of the tumor cells, was interpreted as diffusely or focally positive or negative, respectively.

### Fluorescent in Situ Hybridization (FISH)

FISH assays were carried out to detect either the loss of chromosome 3p and chromosome Y or gain of chromosome 7 and 17. Tissue sections were cut from the TMA blocks and deparaffinized. The assays were done using a *VHL*/cen3 probe (ZytoLight® SPEC *VHL*/CEN3 Dual Color Probe, Zytovision,) and centromeric probes for chromosome 7, 17 and Y (Cytocell) according to the manufacturer’s instructions. Slides were digitalized by using a Pannoramic Midi slide scanner (3DHISTECH), and reactions were evaluated using a Pannoramic Viewer (3DHISTECH) in the following way. Fifty tumor cells from each case were examined and were compared with the same number of cells of the peritumoral tissue, which served as an internal control. The cutoff values of chromosomal gain and/or loss were set at the mean ±3SD of the corresponding control values, as done in previous studies [5]. The analysis of 3p deletion was also performed based on a published method [15].

### VHL Gene Sequence Analysis and VHL Gene Promoter Hypermethylation

A PCR-based amplification method was used for *VHL* gene mutation analysis as earlier described [16]. The *VHL* exons were amplified via specific primer pairs (Supplementary Table 2). In the case of pathological mutation, the apparently tumor-free renal tissue was analyzed as well. A GenomeLAB DTCS - Quick Start Kit (Beckman Coulter) was used for DNA sequencing. The latter was carried out according to the manufacturer’s instructions using the GenomeLab GeXP Genetic Analysis System (Beckman Coulter). The methylation status of *VHL* gene promoter region was determined using the methylation-specific PCR method. The extracted genomic DNA was modified using the EpiJET Bisulfite Conversion Kit (ThermoFischer Scientific), and followed by PCR-based amplification with methylation-specific primer pairs (Supplementary Table 3). The methylation status (non-methylated, methylated) was determined by gel electrophoresis of the PCR products, as reported previously [17].

### Criteria for Diagnosing a Tumor as CCPRCC

The diagnosis of CCPRCC was made if the above-mentioned morphology together with characteristic immunophenotype (CK7- and CA9-positivity, negative CD10 or at most focal CD10-positivity, negative TFE3 and TFEB stainings), along with the lack of genetic alterations indicating CCRCC (3p deletion, *VHL* mutation, *VHL* promoter hypermethylation), and PRCC (7 and 17 trisomy, loss of Y) were detected. Tumors with the same morphology, CK7 and CA9 coexpression, but with diffuse CD10-positivity or with altered *VHL* status were classed as CCRCC.

## Results

Using the inclusion criteria, we retrieved 31 samples. All tumors coexpressed CK7 and CA9. The TFE3 and TFEB reactions were uniformly negative; and the CD10 and the AMACR reactions were negative in 27 and 30 cases, respectively. The FISH assays for papillary RCC, available in 27 cases, and deletion of chromosome 3p, available in 29 cases, yielded negative results. The histomorphology, the results for *VHL* mutation and *VHL* methylation testing, and the immunophenotype confirmed 21 cases as CCPRCC and 10 cases as CCRCC. The principal characteristics of the two subsets are summarized in Tables 1 and 2*.*

**Table 1:** Clinicopathological features of the cases examined

Patient	Sex	Age (y)	ESRD	Tumor-related symptoms	Size (mm)	AJCC Stage	ISUP Grade	Follow-up period (months)*	Progression§	Comment
Clear cell papillary RCC										
1	M	68	No	No	21	T1aNxMx	1	31	No	
2	M	57	No	No	20	T1aNxMx	2	35	No	
3	M	64	No	No	30	T3aNxMx	2	NA	ND	Sinus fat tissue infiltration
4	F	68	No	No	30	T1aNxMx	1	12	No	
5	M	84	ESRD	No	10	T1aNxMx	1	1	No	
6	F	63	No	No	8	T1aNxMx	2	46	No	Ipsilateral oncocytoma
7	F	81	No	No	25	T1aNxMx	1	113	No	
8	F	78	No	No	20	T1aNxMx	1	184	No	
9	M	56	ACKD	No	10	T1aNxMx	1	85	No	
10	M	66	No	No	11	T1aNxMx	1	3	No	
11	F	49	No	No	38	T1aNxMx	1	10	No	
12	M	75	No	No	65	T1bNxMx	2	80	No	
13	F	52	No	No	25	T1aNxMx	2	158	No	
14	M	32	No	No	8	T1aNxMx	1	101	No	
15	F	57	No	No	6	T1aNxMx	1	NA	ND	
16	F	30	ESRD	No	8	T1aNxMx	1	86	No	
17	F	60	No	Abdominal pain	22	T1aNxMx	1	3	No	
18	M	76	No	No	10	T1aN0Mx	1	62	No	Ipsilateral angiomyolipoma and papillary adenomas
19	F	69	No	No	13	T1aNxMx	1	8	No	
20	F	28	ESRD	No	20	T1aNxMx	2	59	No	Tumor in a graft kidney
21	F	56	No	No	30	T1aNxMx	1	NA	ND	
Clear cell RCC										
22	F	41	No	No	20	T1aNxMx	1	26	No	
23	F	44	No	No	50	T1bN1Mx	1	36	No	Lymph node metastasis
24	M	37	No	Hematuria	37	T1aNxMx	1	67	No	Contralateral clear cell RCC two months later
25	M	47	ACKD	No	19	T1aNxMx	1	12	No	
26	F	53	No	No	30	T1aNxMx	1	19	No	
27	F	69	No	No	24	T1aNxMx	2	37	No	
28	M	69	No	Lumbar pain	15	T1aNxMx	1	100	No	Ipsilateral papillary adenoma
29	M	40	No	No	30	T1aNxMx	1	10	No	
30	F	51	No	No	40	T1aNxMx	1	3	No	
31	M	61	No	No	25	T1aNxMx	2	6	No	

*Follow-up, determined from the surgery to the last follow-up; §Progression, assessed by radiological and/or autopsy data

*M* male; *F* female; *ESRD* end-stage renal disease; *ACKD* acquired cystic kidney disease; *NA* not available; *ND* no data

**Table 2:** Morphological, immunohistochemical, and molecular characteristics of the cases examined.

Patient	Architecture of tumor volume (%)					Immune profile (%)					Molecular characteristics					
	Tubular	Papillary	Cystic	Solid	LiN	CK7	CA9	CA9 cup-shaped	CD10	AMACR	+7	+17	-Y	-3p	*VHL* mut	*VHL* met
Clear cell papillary RCC																
1	90	-	-	10	No	Diff	Diff	Yes	Neg	Neg	-	-	-	-	wt	ua
2	88	2	-	10	Yes	Diff	Diff	Yes	Foc	Neg	-	-	-	-	wt	-
3	95	-	-	5	Yes	Diff	Diff	Yes	Neg	Neg	nd	nd	nd	nd	nd	nd
4	59	1	20	20	Yes	Diff	Diff	Yes	Neg	Neg	-	-		-	wt	-
5	100	-	-	-	Yes	Diff	Diff	Yes	Neg	Neg	-	nd	-	-	nd	nd
6	95	5	-	-	Yes	Diff	Diff	Yes	Foc	Neg	-	-		-	wt	-
7	44	5	50	1	No	Diff	Diff	Yes	Neg	Neg	-	-		-	wt	-
8	50	50	-	-	Yes	Diff	Diff	Yes	Neg	Neg	-	-		-	ua	-
9	80	10	-	10	No	Diff	Diff	Yes	Neg	Neg	-	-	-	-	wt	-
10	95	5	-	-	Yes	Diff	Diff	Yes	Neg	Neg	-	-	-	-	wt	-
11	50	40	10	-	Yes	Diff	Diff	Yes	Neg	Neg	-	-		-	wt	-
12	50	-	50	-	Yes	Diff	Diff	Yes	Neg	Neg	-	-	-	-	*5’UTR SNP*^*#*^	-
13	-	80	20	-	No	Diff	Diff	Yes	Neg	Neg	-	-		-	ua	-
14	80	10	10	-	Yes	Diff	Diff	Yes	Neg	Poz^§^	-	-	-	-	wt	-
15	-	20	80	-	Yes	Diff	Diff	No	Neg	Neg	-	-		-	ua	nd
16	95	-	-	5	Yes	Diff	Diff	No	Neg	Neg	-	-			ua	nd
17	50	20	30	-	No	Diff	Diff	No	Neg	Neg	-	-		-	wt	-
18	90	-	5	5	Yes	Diff	Foc	Yes	Neg	Neg	nd	nd	nd	nd	nd	-
19	45	1	50	4	Yes	Diff	Foc	Yes	Neg	Neg	-	-		-	nd	-
20	89	-	1	10	Yes	Diff	Foc	Yes	Neg	Neg	-	-		-	ua	-
21	85	1	5	10	Yes	Diff	Foc	No	Neg	Neg	-	-		-	ua	-
Clear cell RCC																
22	50	5	35	10	Yes	Diff	Diff	Yes	Neg	Neg	-	-		-	*mut*^*a*^	+
23	50	20	30	-	Yes	Diff	Diff	Yes	Diff	Neg	nd	nd		-	*mut*^*b*^	nd
24	10	50	20	20	No	Diff	Diff	No	Diff	Neg	-	-	-	-	*5’UTR SNP*^*¶*^	-
25	80	10	10	-	No	Diff	Diff	Yes	Neg	Neg	-	-	-	-	wt	+
26	95	1	-	4	Yes	Diff	Diff	Yes	Neg	Neg	-	-		-	wt	+
27	20	10	70	-	Yes	Diff	Diff	Yes	Neg	Neg	-	-		-	wt	+
28	40	-	60	-	Yes	Diff	Diff	Yes	Neg	Neg	-	-	-	-	ua	+
29	20	40	40	-	Yes	Diff	Diff	No	Neg	Neg	-	-	-	-	wt	+
30	30	-	20	50	No	Diff	Foc	No	Neg	Neg	-	-		-	wt	+
31	90	-	-	10	Yes	Diff	Foc	No	Neg	Neg	-	-	-	-	*mut*^*c*^	nd

*LiN* linear nuclear arrangement from basement membrane; *+7 and +17* trisomy of chromosome 7 and 17, respectively; *-Y* deletion of chromosome Y; *-3p* deletion of chromosome 3p; *VHL* mut, von Hippel-Lindau gene mutation status; *VHL* met, von Hippel-Lindau gene methylation status; *nd* not determined; *wt* wild type; *ua* unsuccessful analysis; *5’UTR SNP* single nucleotide polymorphism in 5’ untranslated region; *diff* diffuse; *foc* focal; *neg* negative (less than or equal to 10%)

^§^ weak granular positivity; ^#^ exon 3 could not be amplified; ^¶^ exon1b could not be amplified; ^a^ c.221T>A/p.V74N; ^b^ c.625C>T/p.G209*; ^c^ c.354_361delCTTCAGAGinsT

### Features of CCPRCCs

Here, 21 tumors were examined, and the specimens were obtained from 12 females and 9 males. The mean age was 60 years (with range 28 to 84 years). Partial nephrectomy was performed in 4 patients and radical nephrectomy in 17 patients; and one tumor developed in a transplanted kidney. Twenty cases were incidental findings of imaging performed for non-urological symptoms.

### Gross Findings

All the tumors were solitary, and the mean size was 23 mm (with range 6 to 65 mm). Cystic change was present in 12 samples; and multilocular cystic mass existed in cases 7 and 15. The tumorous parenchyma was grey-white to yellow-brown, occasionally with small hemorrhagic foci.

### Microscopic Findings

**Fig. 1:**
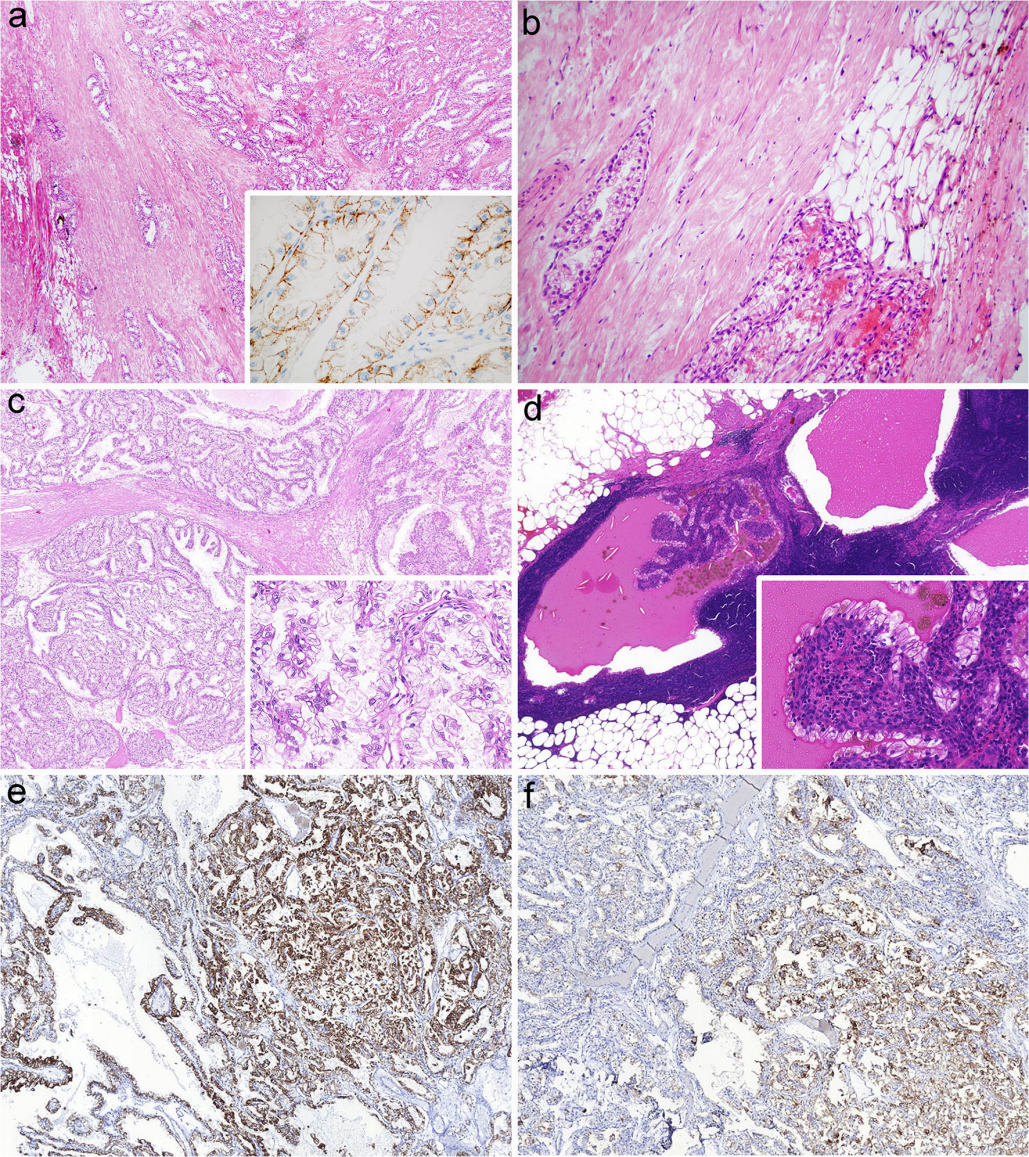
**a-b Case 3 with sinus fat invasion** The tumor showed a branching tubular pattern and an immunophenotype characteristic for CCPRCC (basolateral CA9 reaction in the insert) (**a**). Superficial infiltration of sinus fat was seen (**b**). **Figure1c-f Case 23 with lymph node metastasis** The tumor displayed the morphological features of CCPRCC, partly with papillary architecture (**c**). The lymph node metastasis was mainly cystic; with some papillary infoldings (insert in figure d) (**d**). The tumor exhibited a diffuse CK7 positivity (**e**), but extensive CD10 staining was also observed (**f**). The latter, together with the *VHL* gene mutation detected in this tumor were not consistent with the diagnosis of CCPRCC

Each tumor was circumscribed, and at least one thin fibrous, or fibromuscular capsule was present, except in one case. The capsule was thick (400-800 μm) and contained smooth muscle in 13 tumors. A minimal infiltration of renal sinus fat was observed in Case 3 (Fig. 1a, b). Vascular invasion was not detected in any of the cases examined. The dominant growth pattern was tubulo-acinar (15 tumors), with cyst formation in a continuum from microscopic to macroscopic cystic spaces in 12 samples. Also, a papillary architecture was observed in 14 tumors and was detected mainly focally, except in one case where it was predominantly seen. Solid areas with compact cell nests and trabeculae were seen in 11 samples, and it was mostly made up of a small proportion of the tumorous parenchyma. Foamy macrophages, psammoma bodies and necrosis were absent. Although the clear cell phenotype was a distinctive feature, the tumor cells displayed various cytological characteristics. Most of them were cuboidal or columnar, but flattened forms in cysts, as well as elongated cells arranged focally in a fascicular pattern in solid areas were also encountered. Some tumor cells – especially in solid areas – had eosinophilic cytoplasm. Prominent nucleoli were not present. The linear arrangement of nuclei together with its orientation away from the basement membrane was observed in 16 tumors (from a minimal to an extensive presence). Mitotic figures were occasionally seen. Stromal smooth muscle was found in 18 cases including two samples with abundant myomatous stroma (cases 1 and 7).

### Immunohistochemical and Molecular Profile

All tumors exhibited a strong and diffuse CK7 expression. Immunoreaction for CA9 resulted in diffuse staining in 17 tumor samples, and focal staining in 4 tumor samples. The “cup-shaped” pattern was detected in 17 cases, visible mainly in the tubular and cystic areas. Focal CD10-positivity was found in 2 samples. Weak granular, diffuse AMACR-positivity was noted in Case 14.

The mutation status of the *VHL* gene was investigated in 11 samples, and in Case 12, single nucleotide polymorphism (SNP) in untranslated region (UTR) was found. The *VHL* gene promoter hypermethylation status was analyzed in 16 samples; and none of these harbored promoter region hypermethylation.

### Follow-up

The median time was 52.5 months (with range 1 to 184 months). Three patients did not have a follow-up, and two patients died in non-cancer-related causes. No evidence of tumor progression and recurrence was documented in the data of the 18 surviving patients.

#### Features of CCRCCs Mimicking CCPRCC

In this group, we analyzed 10 cases, and the mean age was 51 years (with range 37 to 69 years) with 5 female and 5 male patients. Half of the cases were treated via partial nephrectomy. Tumor-related symptoms were registered in two patients. In Case 23, a CCRCC (CA9 and CD10: diffusely positive; CK7 negative) was resected from the contralateral kidney two months after the first surgery. And in Case 24, a metastatic perihilar lymph node was removed together with the tumorous kidney (Fig. 1c-f).

### Gross Findings

The mean size of the tumors was 29 mm (with range 15 to 50 mm). Cystic change was noticed almost in all cases (9/10). The cut surface was indistinguishable from those seen in CCPRCC.

### Microscopic Findings

A capsule containing smooth muscle was present in 6 cases. The predominant growth pattern was tubulo-acinar (5/10), followed by cystic (2/10), papillary (1/10) and solid (1/10). In Case 29, the distribution of tubulo-acinar, papillary and cystic pattern was the same. The tumors were composed of clear cytoplasm cells with focal eosinophilic granulations. Also, an apical linear nuclear arrangement was noted in 6 cases, and 2 cases contained a smooth muscle rich stroma. The infiltration of the renal vein, renal sinus and perinephric fat tissue was not observed.

### Immunohistochemical and Molecular Profile

There was a coexpression of CK7 in a diffuse fashion and CA9 in a diffuse (8 cases) or focal fashion (2 cases). The cup-shaped distribution of CA9 was present in 6 cases. Diffuse CD10-positivity was observed in cases 23 and 24.

**Fig. 2:**
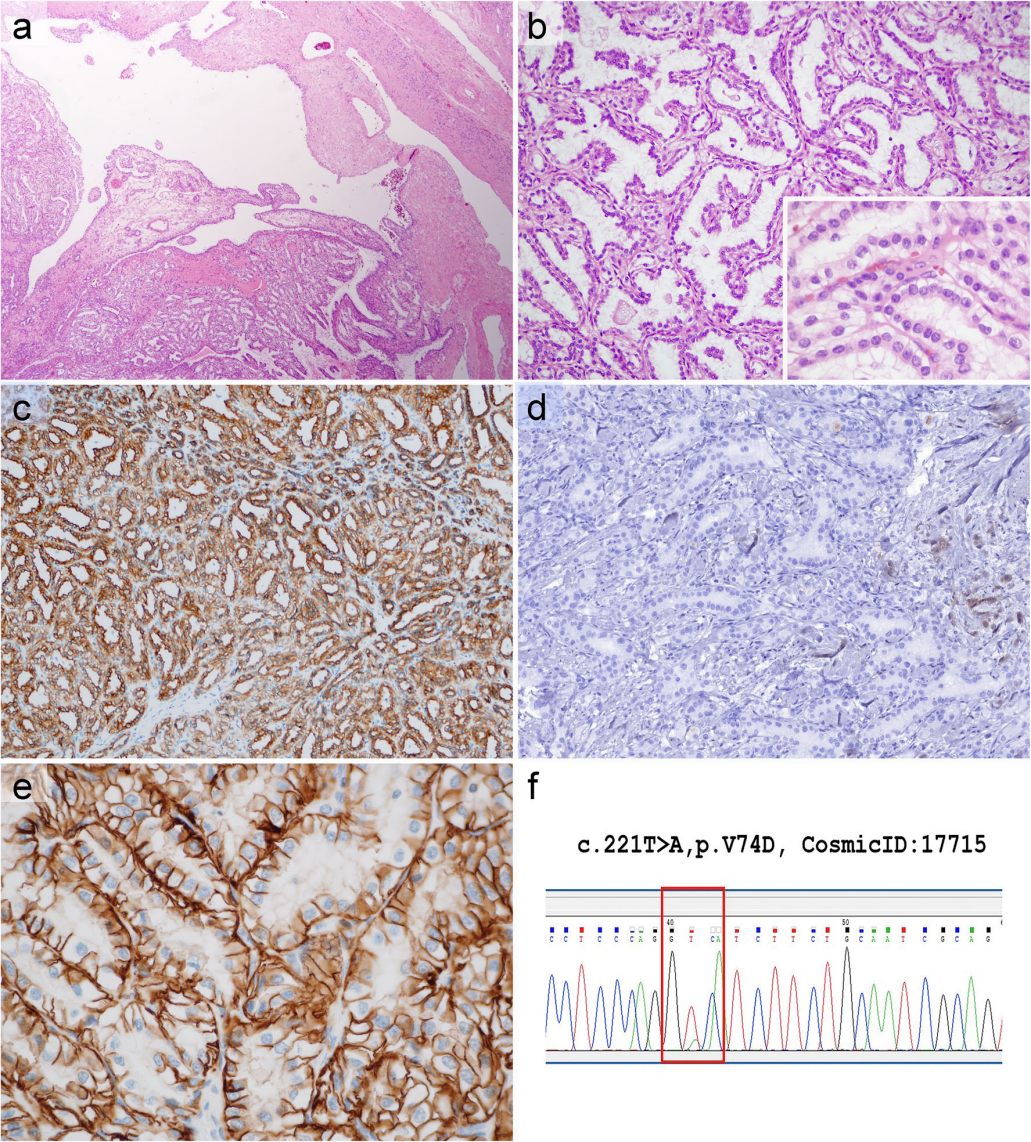
**Case 22 exhibiting morphology and immunophenotype completely consistent with CCPRCC, but containing a*VHL*gene mutation.** The tumor had a thick fibromyomatous capsule and it was composed of both solid and cystic areas (**a**). Branching tubular architectural pattern was the most characteristic (**b**). The tumor cells were diffusely positive for CK7 (**c**); and negative for CD10 (**d**). CA9 immunoreaction also resulted in a diffuse staining with a basolateral pattern (**e**). *VHL* gene mutation was detected by direct sequencing (**f**)

The *VHL* gene mutation status was analyzed in 9 samples, and in cases 22, 23 and 31 a pathogenic mutation was identified that was not present in the tumor-free renal parenchyma (Fig. 2). The sequencing revealed an SNP without any clinical significance in Case 24. The remaining 5 tumors analyzed harbored no genetic change. Also, the *VHL* gene promoter hypermethylation was tested in 8 cases, and 7 of them possessed promoter region hypermethylation.

### Follow-up

All the cases had follow-up data with a median of 31.6 months (with range 3 to 100 months). None of them experienced any recurrence and cancer-related death.

## Discussion

The WHO classification of RCCs defines the subsets via a synthesis of histopathological, immunohistochemical, and genetic data [1]. In the current study, we focused on the discrimination of CCPRCC from other tumor types by applying the well-known immunohistochemical markers supplemented with a molecular analysis that seeks to find chromosomal abberations and *VHL* abnormalities (including mutations as well as methylation analysis). We made a formal diagnosis of CCPRCC when both the immunohistochemical and the genetic tests were in complete accordance with the histology.

All the RCC subtypes with clear cell phenotype (i.e. *TFE3* or *TFEB* translocation RCCs, *TCEB1*-mutated RCC [18], RCC with 8p monosomy [19], RCC with prominent smooth muscle stroma (RCCSMS) [20–25] and RCC associated with von Hippel-Lindau syndrome) can exhibit a CCPRCC-like histomorphology [26, 27], but CCRCC cases pose the biggest difficulty because this tumor type is the most common and it also has some morphological similarities. CCRCCs are viewed as tumors that are CA9+ and CD10+, and display no more than a focal CK7 positivity. In contrast, the immunophenotype of CCPRCCs is CK7+, CA9+, and CD10-. Perhaps diffuse and strong CK7 positivity is considered the most important and an obligatory diagnostic criterion for CCPRCC. Nevertheless, the lack of a widespread CD10 reaction is also required.

We performed our case selection based on histological features suggestive of CCPRCC, and all the tumors displayed diffuse CK7 staining. In two of them, however, CD10 positivity was also diffuse, which supported our diagnosis of CCRCC. In another subset of our cases, the morphology and immunophenotype wholly favoured the diagnosis of CCPRCC; however, either *VHL* mutation (3 cases) or *VHL* promoter hypermethylation (7 cases) was present. We accepted the view of Hes et al. who recommended not classifying cases with any *VHL* gene abnormality as CCPRCC [28]. Based on their approach, our tumors with altered *VHL* status were classified as CCRCC.

After performing an immunohistochemical and molecular analysis, our selected cases with histology of CCPRCCs were subdivided into two groups. These are CCPRCCs (21 cases), and CCRCCs with diffuse CK7 positivity (10 cases).

### Features of CCPRCC Cases

The characteristic pattern of these tumors was branching tubulo-acinar that was commonly accompanied by cyst formation. Papillary areas, however, were detected as a minor component except in three cases. As similar findings on the extent of papillarity were obtained by Aydin et al. [5] and by Williamson et al. [29], we conclude that CCPRCC with predominant papillarity probably occurs quite rarely. Therefore, the WHO designation of this entity seems inaccurate, and the appellation “tubulopapillary” would perhaps be more apt, as was suggested by Aydin et al [5].

Linear nuclear arrangement away from the basement membrane is regarded as characteristic for CCPRCC [30]. Actually, 16/21 of CCPRCCs and 7/10 CCRCCs with diffuse CK7-positivity harbored this phenomenon (from a minimal to extensive presence). Dhakal et al. examined 37 tumors with a morphologic overlap between CCPRCC and CCRCC features, and linear nuclear arrangement was not the exclusive feature of cases classified as CCPRCC [31]. In another series of CCPRCC, Williamson at al. noticed linear nuclear arrangement only in 24/55 cases [32]. These findings suggest that linear nuclear arrangement is an overemphasized phenomenon, since its absence does not exclude the possible diagnosis of CCPRCC, and its presence does not necessarily support the diagnosis of CCPRCC.

The cup-shaped expression of CA9 was not uniformly present in our series. A diffuse cup-shaped expression was observed in 17/21 samples, while a dominant box-shaped staining with a focal cup-shaped expression was noted in 4/21 samples. Upon reviewing the literature, a cup-shaped expression involving 50% of tumor cells was reported by Rohan et al. in 3 out of their 9 cases [6]; and Dhakal et al. noted a cup-like expression of CA9 in 74% of their cases [31]; and Aydin et al. did not mention this feature at all in their 36 cases [5]. Since in our experience the cup-shaped staining pattern cannot be discerned unambiguously in solid areas, the absence of cup-shaped expression should be interpreted with caution when making a concrete diagnosis of CCPRCC for a specific case. A diffuse and weak granular AMACR-positivity was seen in one case. We reviewed the immunoprofile of the published CCPRCC cases and, albeit rarely, AMACR-positivity was reported [5, 6, 11, 29, 32–34]; hence if it is present, it does not necessarily contradict the diagnosis of CCPRCC. Focal CD10-positivity was encountered in two, otherwise completely typical CCPRCC, and *VHL* gene abnormalities were not present in these samples. The focal extent of CD10 expression may indicate the possibility of RAT, because a lack of a cystic component viewed microscopically, and the triple coexpression of CK7, CA9 and a certain degree of CD10 were noted in a series of RCC cases classified as RAT [35].

Our CCPRCC group comprised 19 pT1a, 1 pT1b and 1 pT3a cases, respectively. To our knowledge, ours is the first reported case with infiltration outside of the kidney parenchyma. Also, in Case 20 the tumor developed in a transplanted kidney. In a recently published review, Dhakal at al. [36] summarized the findings of 24 articles that reported tumors in transplanted kidneys, but among the 48 tumors described, not one was CCPRCC. Coexisting benign tumors and CCPRCC were observed in two cases. Actually, in Case 6 the oncocytoma had been detected clinically, and during the grossing CCPRCC was discovered. All of our CCPRCC cases had an excellent clinical outcome, reinforcing the view that the carcinoma designation might be exaggerated [14, 37, 38].

### Features of CCRCCs with Diffuse CK7-Positivity

A series of CCRCC with diffuse CK7-posivity was published a decade ago by Mai et al [39]. Similar to our experiences, these samples were small-sized, and a non-metastatic course was recorded over a mean of a 3-year follow-up; and diffuse CK7-positivity was viewed as the indicator of indolent behaviour [40].

Our results provide further clinicopathologic data on this rare subset of CCRCC. Accordingly, neither 3p deletion, nor other chromosomal anomalies were present. The *VHL* gene sequence analysis revealed pathologic mutations in cases 22, 23 and 31. Since *VHL* mutations were not identified in the non-tumorous renal tissue, the possibility of VHL-disease-associated CCRCC was excluded.

In seven samples, the histological and immunphenotypic data favoured the diagnosis of CCPRCC; however, the presence of the *VHL* gene promoter hypermethylation abnormality leads us to place these samples into the CCRCC group. In the study of Herman et al. on silecing of the *VHL* gene by DNA methylation, the hypermethylation of a CpG island in the 5’ region was noted in 5 samples out of 26 CCRCCs [16]. Four of these had lost one copy of *VHL,* while one retained two heavily methylated alleles. The latter observation indicated that hypermethylation may inactivate the *VHL* gene even when both wild-type alleles are retained [16]. In our analysis, hypermethylation was noted in seven cases; moreover coexisting *VHL* gene mutation and methylation was seen in Case 22. After a search for methylation data, only 2 tumors analyzed were found in the literature out of 400 or so CCPRCCs [5, 14]. Methylation analyses performed by others in the future may validate our assumption that a *VHL* promoter hypermethylation is definitely not compatible with the diagnosis of CCPRCC. In Case 24 (and in Case 12 in the CCPRCC group) an SNP was observed in the 5’ UTR region, a finding treated as insignificant, because the nucleotide change did not induce any amino acid change as well. Interestingly, in 8 cases the histological and immunphenotypic data were entirely consistent with the histopathological diagnosis of CCPRCC, but the presence of *VHL* abnormalities led us to place these samples into the group of low-grade CCRCC with CK7 immunoreactivity and no 3p loss. Every case was in the pT1 stage, and there was no progression or recurrence.

In summary, in our study the immunophenotype and the genetic profile of 31 RCCs composed of clear cells, low-grade nuclei and a tubulopapillary architecture were investigated retrospectively. Twenty-one cases were classified as CCPRCC (CK7+, CA9+; -3p absent, *VHL* abnormality not present) and 10 as CCRCC with diffuse CK7-positivity (CK7+, CA9+; -3p absent, *VHL* abnormality present). Based on our findings, the following conclusions can be drawn. First, CCPRCCs rarely exhibit a predominant papillary architecture, hence their name is misleading. Second, a linear nuclear arrangement away from the basement membrane and cup-like CA9 positivity are not obligatory features. Third, the evidence for their malignant potential is still subject of debate. Fourth, RCCs with CCPRCC morphology, diffuse CK7 positivtiy, and with an altered *VHL* status (mutation, or promoter hypermethylation) do exist; and these tumors can be interpreted as CCRCC with diffuse CK7 positivity, and they can be definitely differentiated from CCPRCCs only by carrying out molecular tests for the *VHL* status. And last but, not least the biological behavior of both CCPRCCs and CCRCCs with diffuse CK7 positivity seems to be indolent with a favorable clinical outcome. Overlapping and discriminating features of CCPRCCs and CCRCCs are summarized in Table 3.

**Table 3:** Overlapping and discriminating features of CCRCCs and CCPRCCs. As we accepted the view of Hes et al. [18] that *VHL* gene alteration is not compatible with the diagnosis of CCPRCC, altered *VHL* status was found as the most reliable discriminating feature between CCRCCs and CCPRCCs in our cohort

	Tubulopapillary architecture	Subnuclear vacuolization	Stromal SM	Diffuse CK7+	Diffuse CD10+	CA9 cup-shaped	-3p	*VHL* mut	*VHL* met
CCCRCC	+/-	+/-	+/-	+/-	+/-	+/-	+/-	+/-	+/-
CCCPRCC	+	+/-	+/-	+	-	+/-	-	-	-

*SM* smooth muscle; *mut* mutation; *met* hypermethylaiton

## Electronic supplementary material

ESM 1 (DOCX 13 kb)
