# Supplementary material for: Renal Cell Carcinoma with Clear Cell Papillary Features: Perspectives of a Differential Diagnosis
Source: Pathol Oncol Res. 2019 Oct 26;26(3):1767–76. doi: 10.1007/s12253-019-00757-3 (PMC7297853; doi:10.1007/s12253-019-00757-3)
Supplement: Supplementary file 1 — (DOCX 13 kb) [file 12253_2019_757_MOESM1_ESM.docx]

**Supplementary Materials**

Supplementary Table 1

Details of the antibodies used for immunohistochemistry analysis.

| Antibody | Source | Clone | Dilution |
| --- | --- | --- | --- |
| CA9 | Novus Biologicals | polyclonal | 1:4000 |
| CK7 | Cell Marque | OV-TL 12/30 | 1:100 |
| CD10 | Biocare Medical | CM129 | 1:50 |
| AMACR | Abcam | polyclonal | 1:100 |
| TFEB | Bioss USA | polyclonal | 1:50 |
| TFE3 | Cell Marque | MRQ-37 | 1:100 |

Supplementary Table 2

Primer sequences for the sequencing of the *VHL* gene. The Exon 1 was split into two parts.

| *VHL* gene | Forward Primer | Reverse Primer |
| --- | --- | --- |
| Exon 1a | 5’- AGCGCGTTCCATCCTCTAC- 3’ | 5’-CTGCGATTGCAGAAGATGAC -3’ |
| Exon 1b | 5’- TACGGCCCTGAAGAAGACGG -3’ | 5’- GGGCTTCAGACCGTGCTATC -3’ |
| Exon 2 | 5’- AGGACGGTCTTGATCTC-3’ | 5’- GATTGGATAACGTGCCTGAC -3’ |
| Exon 3 | 5’- GTTGGCAAAGCCTCTTGTTC -3’ | 5’- GAAGGAACCAGTCCTGTATC -3’ |

Supplementary Table 3

Primer sequences for the methylation-specific PCR analysis of the *VHL* gene.

| *VHL* gene | Forward Primer | Reverse Primer |
| --- | --- | --- |
| Methylated | 5’-TGGAGGATTTTTTTGCGTACGC-3’ | 5’-GAACCGAACGCCGCGAA -3’ |
| Unmethylated | 5’-GTTGGAGGATTTTTTTGTGTATGT-3’ | 5’-CCCAAACCAAACACAACAAA-3’ |
|  |  |  |
|  |  |  |
